# Chemical Composition, Biocompatibility, and Anti-*Candida albicans* Activity of *Schinus weinmanniifolia* Mart. ex Engl.

**DOI:** 10.3390/pathogens14080799

**Published:** 2025-08-09

**Authors:** João Andrade, Adriana Almeida-Apolonio, Fabiana Dantas, Cláudio Nogueira, Luciano Pinto, Carlos Moraes, Liliana Fernandes, Maria Elisa Rodrigues, Mariana Henriques, Kelly Oliveira

**Affiliations:** 1 Faculdade de Ciências da Saúde, Universidade Federal da Grande Dourados, Dourados 79804-970, MS, Brazil; victorandrade.j.s@gmail.com; 2 Curso de Engenharia Ambiental e Sanitária, Universidade Estadual do Mato Grosso do Sul, Dourados 79804-970, MS, Brazil; aaraujo.a@hotmail.com; 3 Faculdade de Ciências Biológicas e Ambientais, Universidade Federal da Grande Dourados, Dourados 79804-970, MS, Brazil; fabianasilva@ufgd.edu.br; 4 Faculdade de Ciências Exatas e Tecnologia, Universidade Federal da Grande Dourados, Dourados 79804-970, MS, Brazil; claudiornogueira@ufgd.edu.br; 5 Departamento de Alimentos e Nutrição Experimental, Faculdade de Ciências Farmacêuticas, Universidade de São Paulo, São Paulo 05508-000, SP, Brazil; lucianosilva58@gmail.com; 6 Departamento de Química, Centro de Ciências Exatas e de Tecnologia, Universidade Federal de São Carlos, São Carlos 13565-905, SP, Brazil; andre453_@hotmail.com; 7 FRG-Fungi Research Group, Centre of Biological Engineering, University of Minho, Campus de Gualtar, Braga 4710-057, Portugal; lilianafernandes@ceb.uminho.pt (L.F.); elisarodrigues@ceb.uminho.pt (M.E.R.); mcrh@deb.uminho.pt (M.H.); 8 LABBELS—Associate Laboratory, Braga/Guimarães 4710-057, Portugal

**Keywords:** *Schinus weinmanniifolia*, phytochemicals, vulvovaginal candidiasis, antifungal activity

## Abstract

Recurrent vulvovaginal candidiasis (RVVC), predominantly caused by *Candida albicans*, represents a global health issue, particularly in developing regions. This study explores the antifungal potential of aqueous leaf extract of *Schinus weinmanniifolia* Mart. ex Engl., a native Latin American plant. The extract was evaluated for phytochemical composition, antifungal efficacy, and safety profile. Phytochemical analyses identified six major compounds, including shikimic acid, gallic acid, and methyl gallate, with antioxidant and antimicrobial properties. The extract showed potent antioxidant activity, with IC_50_ values between 1.52–5.51 µg/mL. It strongly inhibited *C. albicans*, with a minimum inhibitory concentration (MIC) of 1.95 µg/mL, and was active against other yeasts (MIC 0.48–62.5 µg/mL). The growth kinetics assay revealed reduced *C. albicans* viability after 12 h at 2 × MIC versus the positive control. Scanning electron microscopy confirmed reduced fungal counts without morphological damage. The extract impaired *C. albicans* virulence, reducing germ tube formation by 75.49% and hyphal transition by 84.34%, outperforming fluconazole. Biocompatibility assays showed it is non-hemolytic (IC_50_ > 1000 µg/mL), non-mutagenic, and highly selective for fungal cells (SI = 512.82), suggesting minimal human cell toxicity. In conclusion, the extract combines strong antifungal activity and favorable safety, with cost-effective preparation suitable for traditional medicine in resource-limited regions.

## 1. Introduction

*Candida albicans*, is part of the microbiota of the skin, mucosal surfaces, gastrointestinal tract, and genitourinary tract. However, it is also an opportunistic yeast associated with various types of infections, especially in immunocompromised individuals, in hospital settings, or after the inappropriate use of antimicrobials [1]. Due to its clinical relevance and public health impact, this species has been included in the critical group of the World Health Organization’s priority list of fungal pathogens [2].

Among the various infections caused by *C. albicans*, recurrent vulvovaginal candidiasis (RVVC) stands out as a chronic and potentially debilitating vaginal infection. Worldwide, it affects approximately 138 million women per year and is projected to reach 158 million by 2030 [3]. Latin American countries present particularly high rates of RVVC [3]. In this context, notably, social vulnerability factors, such as inadequate basic sanitation, low socioeconomic status, limited access to healthcare services, and insufficient investment in public policies focused on research and technological innovation, can significantly exacerbate the challenges related to the treatment and control of RVVC in the region [4,5,6,7,8,9,10].

The most commonly used treatment for RVVC involves local or oral administration of the antifungal fluconazole (FLU). However, prolonged use of this drug is associated with high costs and recurrence of symptoms after treatment completion [11]. In this context, the search for feasible alternatives to control the development of *C. albicans* and minimize the progression of RVVC becomes both relevant and urgent. Research on plant extracts as potential antifungal alternatives in the treatment of RVVC reflects the ethnopharmacological knowledge of different populations [12,13,14,15] and indicates promising avenues for new therapeutic approaches. It is also noteworthy that only 6% of the more than 300,000 higher plant species have been thoroughly investigated regarding their biological properties [16].

In this context, species of the genus *Schinus* L. stand out due to their extensive traditional use and biological properties already described in the literature [17]. The species *Schinus terebinthifolius* Raddi, the most studied of the genus, is widely used in folk medicine, especially in the treatment of ‘women’s diseases,’ with application in sitz baths postpartum, due to its anti-inflammatory and healing properties [18]. Studies report its antioxidant, anti-inflammatory [19,20], and antimicrobial potential [21,22,23,24,25], effects that may be associated with the presence of chemical compounds such as ethyl gallate, methyl gallate, quercitrin, myricetrin, rutin, ferulic acid, and gallic acid [25,26].

*Schinus weinmanniifolia* Mart. ex Engl., a member of the same genus, commonly known as low ‘aroeira’ or field ‘aroeira’, is a fast-growing subshrub, with a height ranging from 0.2 to 1.5 m. One of its main morphological characteristics is the presence of a xylopodium (underground stem), which provides resistance to fire, removal of the aerial part, and nutrient-poor soils [27]. This species is native to Latin American countries such as Brazil, Argentina, Paraguay, and Uruguay [27,28]. Despite its wide distribution, there are few records of its popular use. Some reports from the early 2000s mention its applications as an analgesic, astringent, and emmenagogue [27,29].

Still within this scope, the scientific investigation of *S. weinmanniifolia* is as limited as the records of its popular use. To date, there are only three published studies on the biological activities of the leaves of this species, which have investigated the antioxidant potential of the methanolic extract and its fractions [30], the antimicrobial activity of the essential oil [31], and the antimicrobial activity of the ethanolic extract [32]. To date, no studies have been reported in the literature concerning the biological evaluation of the aqueous extract of this species.

Considering this scientific gap, the pharmacological potential of the species, and the need for new therapeutic options for RVVC, the present study aimed to evaluate the antifungal activity of the aqueous extract of *S. weinmanniifolia* leaves against *C. albicans*, including clinical isolates from cases of vulvovaginal candidiasis (VVC). Additionally, the study sought to characterize its phytochemical and biocompatibility profiles, highlighting its potential as a novel alternative for use in traditional medicine. This species presents itself as a promising therapeutic alternative for the management of RVVC, especially in areas where access to conventional treatments is limited, such as Latin American countries.

## 2. Materials and Methods

### 2.1. Plant Material Collection and Preparation of Aqueous Extract of Leaves of Schinus weinmanniifolia Mart. ex Engl. (AES)

Leaves of *Schinus weinmanniifolia* Mart. ex Engl. were collected in the municipality of Dourados–Mato Grosso do Sul, near the Cural de Arame and Campo Beli farms (22°11’14” S, 54°55’14” W), in December 2024. The plant was identified by Dr. Augusto Giaretta de Oliveira of Universidade Federal da Grande Dourados. A specimen of this species was deposited in the DDMS-UFGD herbarium under registration number 8856. It is registered with the Sistema Nacional de Gestão do Patrimônio Genético e do Conhecimento Tradicional Associado (SisGen) under registration A9BE079.

The collected leaves were dried in a circulation oven at 40 °C for 72 h, under controlled temperature and humidity conditions. After drying, the plant material was crushed and extracted using 10 g of dry plant per 100 mL of water boiling water under stirring for 5 h (to allow cooling to room temperature). Subsequently, the extract was filtered through a 0.45 µm pore size filter paper using a vacuum filtration system, followed by freeze-drying. The lyophilized extract was collected and stored at 4 °C in amber glass vials, tightly sealed and protected from light and moisture. The extraction procedure was performed in triplicate (n = 3), with each replicate processed independently to ensure reproducibility.

### 2.2. Chemical Characterization

The analyses by UHPLC-ESI-MS/MS were performed using an Agilent 6545 Q-TOF LC/MS system (Agilent Technologies, Santa Clara, California, United States of America) and an Agilent Zorbax Eclipse Plus C18 column (2.1 × 50 mm, 1.8 µm) (Agilent Technologies, Santa Clara, CA, USA). The sample (AES) was prepared by weighing the lyophilized extract, dissolving it initially at 1 mg/mL, and then diluting it to a final concentration of 200 ppm in a CH_3_OH-H_2_O (1:1, *v*/*v*) mixture, and subsequently filtered through a 0.22 µm polytetrafluoroethylene (PTFE) membrane filter prior to injection. Acetonitrile (Merck KGaA, Darmstadt, Germany), methanol (Merck KGaA, Darmstadt, Germany), water (Merck KGaA, Darmstadt, Germany), and formic acid (Thermo Fisher Scientific, Waltham, MA, USA), all of HPLC grade, were used in these hyphenated analyses. Column temperature, injection volume, and flow rate were set as 40 °C, 5 μL, and 0.3 mL/min, respectively. The mobile phase was composed of water containing 0.1% (*v*/*v*) of formic acid (solvent A) and acetonitrile (solvent B), and the elution program used was as follows: 5–70% solvent B for 0–10 min (linear gradient); 70–90% solvent B for 10–18 min (linear gradient); 90–100% solvent B for 18–20 min (liner gradient); 100% solvent B for 20–26 min.

The high-resolution mass (HRMS) and MS/MS spectra, which were obtained in the positive and negative ionization modes, were collected in the scan range of mass-to-charge ratio (*m/z*) of 100–1500, using an acquisition rate of 3 spectra per second for both MS and MS/MS data. Three collision energy levels were employed for the acquisition of MS/MS data: High energy [100–500 Da (10–35 ev); 500–1000 Da (35–40 ev); and 1000–1500 Da (40–50 ev)]; medium energy [100–500 Da (8–25 ev); 500–1000 Da (25–35 ev); and 1000–1500 Da (35–45 ev)]; and low energy [100–500 Da (5–15 ev); 500–1000 Da (15–25 ev); and 1000–1500 Da (25–30 ev)]. Dry gas temperature, skimmer voltage, and nebulizer gas pressure were set as described in a previous publication by Do Prado Schneidewind et al. [33], whereas the sheath gas temperature, sheath gas flow rate, drying gas flow rate, and capillary voltage were set as 350 °C, 11 L/min, 8 L/min, and 3.2 kV, respectively. All MS and MS/MS data were processed using Mass Hunter software, version B.08.00.

### 2.3. Antioxidant Activity

#### 2.3.1. Radical Scavenging 2,2-Diphenyl-1-picrylhydrazyl (DPPH)

A 2,2-diphenyl-1-picrylhydrazyl (DPPH) (Merck KGaA, Darmstadt, Germany) free radical scavenging assay was used according to the descriptions by Xiao et al. [34], with modifications. A methanolic DPPH solution was incubated with the AES at concentrations ranging from 0.24 to 125 μg/mL (serial dilution in 80% methanol). Ascorbic acid (AA) (Merck KGaA, Darmstadt, Germany) and Butylated hydroxytoluene (BHT) (Merck KGaA, Darmstadt, Germany) were used as positive controls, and 80% methanol was used as the solvent control. After 30 min of incubation at room temperature while protected from light, absorbance was measured at 517 nm using a spectrophotometer (Quimis, Diadema, Brazil). The results were expressed as the concentration capable of inhibiting 50% of the DPPH free radical (IC_50_). The experiment was performed in triplicate at two different time points.

#### 2.3.2. Radical Scavenging 2,2-Azino-bis (3-Ethylbenzothiazoline-6-sulfonic Acid) (ABTS)

The 2,2-azino-bis (3-ethylbenzothiazoline-6-sulfonic acid) (ABTS) (Merck KGaA, Darmstadt, Germany) free radical scavenging assay was used according to the descriptions by Xiao et al. [34], with modifications. A stock solution of ABTS (7 mM) was prepared with potassium persulfate 16 h before the experiment and kept in the dark at room temperature. The ABTS radical was then diluted in 80% ethanol (Merck KGaA, Darmstadt, Germany) to an absorbance of 730 nm and incubated for 6 min with the AES at concentrations ranging from 0.24 to 125 μg/mL. AA and BHT were used as positive controls, and 80% methanol was used as the solvent control. Absorbance was measured at 730 nm with a spectrophotometer. The results were expressed as the concentration capable of inhibiting 50% of the ABTS free radical (IC_50_). The experiment was performed in triplicate at two different time points.

### 2.4. Antifungal Activity

#### 2.4.1. Microorganisms

Standard yeast strains were obtained from the American Type Culture Collection (ATCC, Rockville, MD, USA): *Candida albicans* (ATCC 90028), used as a reference strain for the antifungal potential and mechanism of action tests, *Candida tropicalis* (ATCC 750), *Candida parapsilosis* (ATCC 22019), *Nakaseomyces glabrata* (*Candida glabrata* ATCC 2001), *Pichia kudriavzeveii* (*Candida krusei* ATCC 6258), *Cryptococcus gattii* (ATCC 56990), and *Cryptococcus neoformans* (ATCC 32045). Given the clinical relevance of vaginal infections caused by *C. albicans*, AES was also evaluated against clinical isolates of *C. albicans* (from VVC). All clinical isolates of *C. albicans* used in this study are deposited in the Coleções Microbiológicas da Rede Paranaense – Taxonline, at the Universidade Federal do Paraná, Brasil, under the following registry numbers: CMRP3475, CMRP3476, CMRP3477, CMRP3478, and CMRP3479. All microorganisms were cryopreserved at −80 °C to ensure viability. For the biological assays, each strain was reactivated in Sabouraud broth (Merck KGaA, Darmstadt, Germany) for 24 h and subsequently subcultured on Sabouraud agar (Merck KGaA, Darmstadt, Germany), followed by incubation for an additional 24 h prior to use in the experiments.

#### 2.4.2. Minimum Inhibitory Concentration (MIC)

The MIC of the extract was determined using the broth microdilution method, following the guideline M27 of the Clinical and Laboratory Standards Institute [35], with adaptations for natural products. Yeast suspensions were prepared in saline solution (0.85% NaCl) (Merck KGaA, Darmstadt, Germany) and standardized to 2.5×10^6^ cells/mL using a spectrophotometer at 530 nm, then diluted 1:50 and 1:20 in RPMI-1640 (Merck KGaA, Darmstadt, Germany). The AES was diluted in RPMI-1640 medium in 96-well microplates to final concentrations of 0.24–1000 μg/mL. FLU (Merck KGaA, Darmstadt, Germany), was used as the standard antifungal. Incubation was conducted at 37 °C for 24 h for *Candida* strains and 37 °C for 48–72 h for *Cryptococcus* strains. Through visual observation, the MIC was determined as the lowest concentration that reduced fungal growth by 50% compared to the positive control (MIC_50_) [36,37]. The experiment was performed in triplicate at two different time points.

#### 2.4.3. Growth Kinetics of *C. albicans*

The growth kinetics assay was conducted with the reference strain *C. albicans* ATCC 90028, as described by Klepser et al. [38], with modifications. The yeast was grown on sabouraud dextrose agar (SDA) and suspended in RPMI-1640 medium, where it was standardized to 2.5×10^6^ cells/mL using a spectrophotometer at 530 nm, and then diluted 1:50 and 1:20 in RPMI-1640. The standardized inoculum was incubated with the MIC, 2 × MIC for AES, and MIC for FLU. The positive control consisted of RPMI-1640 and the inoculum. The suspensions were incubated at 37 °C, and at specific time points (0, 4, 8, 12, 24, 28, 32, 36, and 48 h), aliquots of 10 μL from each treatment were subjected to serial dilutions in saline solution and plated on Sabouraud agar. After 24 h, quantification of Colony Forming Unit per mL (CFU/mL) was performed. The experiment was performed in triplicate at two different time points.

### 2.5. Mechanisms of Action in Planktonic Cells of C. albicans

#### 2.5.1. Scanning Electron Microscopy (SEM)

To observe the morphological changes in the reference strain *C. albicans* ATCC 90028 treated with AES via scanning electron microscopy (SEM), the methodology described by Ramalho et al. [39] was used, with adaptations. Treatments with AES at MIC and 2 × MIC concentrations were carried out in 12-well plates, with an untreated group (inoculated cells without AES) included as a control. After incubation, the culture medium was removed from all wells and washed twice with 1 mL of saline solution (0.85% NaCl). Cells were fixed with 2.5% glutaraldehyde (Merck KGaA, Darmstadt, Germany) for 2 h, after which the solution was aspirated and discarded. Dehydration was followed by the addition of 1 mL of 80% ethanol for 5 min, with subsequent treatments using 90% and 100% ethanol. The microplates were incubated at 30 °C for 12 h for drying. Subsequently, the bottom of each microplate was cut into 1 mm × 1 mm squares corresponding to each well. The samples were then coated with gold and observed under a JSM-6380LV scanning electron microscope (Jeol, Peabody, MA, USA) with images captured at a magnification of 1000×. The experiment was performed in triplicate at two different time points.

#### 2.5.2. Germ Tube Formation (GTF)

To evaluate the activity of AES on germ tube formation (GTF), the descriptions by Haghdoost et al. [40], with adaptations, were considered. For the assay, the reference strain *C. albicans* ATCC 90028 was grown in yeast nitrogen base broth (YNB) (Merck KGaA, Darmstadt, Germany) at 30 °C for 24 h with shaking at 100 rpm, then washed three times with phosphate-buffered saline (PBS) (Merck KGaA, Darmstadt, Germany) and centrifuged at 5000× *g*. The yeast was adjusted to a concentration of 2.5 × 10^6^ cells/mL using a spectrophotometer and diluted 1:50 in RPMI-1640 medium and 1:20 in RPMI-1640 medium containing 10% FBS. Microplates were prepared with MIC concentrations, 2 × MIC for AES, and MIC for FLU. RPMI-1640 containing 10% FBS and the inoculum was used as a positive control, and RPMI without supplementation but with inoculum was used as a negative control. Subsequently, the plates were incubated at 37 °C for 3 h while shaking at 250 rpm. The GTF was observed using an optical microscope (Leica, São Paulo, Brazil) (magnification 400×), and the presence of germ tubes was quantified for every 100 cells (observed in different microscopic fields). Germination reduction percentage (GRP) was calculated using the following equation: GRP = (GTF control − GTF sample)/GTF control × 100. The experiment was performed in triplicate at two different time points.

#### 2.5.3. Yeast-to-Hyphal Transition

The action of AES on the yeast-to-hypha transition of the reference strain *C. albicans* ATCC 90028 was evaluated as described by Bravo-Chaucanés et al. [41], with adaptations. *C. albicans* cells were initially grown in yeast peptone dextrose broth (YPD) (Merck KGaA, Darmstadt, Germany) at 30 °C for 24 h with shaking at 100 rpm. Next, 2 mL aliquots of the overnight cultures were centrifuged at 5000 ×g for 10 min and washed twice with PBS. The cells were then resuspended in PBS, adjusted to an inoculum of 1 × 10^6^ cells/mL using a spectrophotometer, and diluted 1:50 in RPMI-1640 medium or 1:20 in RPMI-1640 medium containing 10% FBS. Cells were exposed to AES at MIC, 2 × MIC, and MIC concentrations of FLU to observe the yeast-to-hypha transition. The positive control was prepared with RPMI-1640 supplemented with 10% FBS and inoculum, and RPMI without supplementation but with inoculum was used as a negative control. Cultures were incubated at 37 °C for 4 h with shaking at 250 rpm. After incubation, hyphal formation was observed using an optical microscope (magnification 400×), and the presence of hyphal formation was quantified for every 100 cells (observed in different microscopic fields). The percentage of hyphal inhibition was calculated using the following equation: Percentage inhibition = (hyphae control–hyphae sample) / hyphae control × 100. The experiment was performed in triplicate at two different time points.

### 2.6. Biocompatibility

#### 2.6.1. Hemolytic Activity

The hemolytic activity of AES was evaluated according to Dhonnar et al. [42], with adaptations. After ethical approval from the Research Ethics Committee of the Universidade Federal da Grande Dourados, Brasil (opinion nº. 5,588,196), 5000 μL of blood were collected in a tube with EDTA (Cral, Cotia, Brazil) from a healthy donor and centrifuged at 1600× *g* at 20 °C for 10 min. The supernatant was discarded, and the pellet was washed three times with PBS by centrifugation at 1600× *g* at 20 °C for 10 min. A 1% erythrocyte solution was prepared for testing.

A quantity of 1000 μL of erythrocyte solution was mixed with 1000 μL of AES at concentrations ranging from 0.24 to 1000 μg/mL. The positive and negative controls were 0.1% Triton-X (Merck KGaA, Darmstadt, Germany) and PBS, respectively. The samples were incubated at 37 °C with shaking at 40 rpm for 60 min, then centrifuged at 1600× *g* at 20 °C for 10 min. The supernatant was measured at 540 nm using a spectrophotometer. The results were expressed as the concentration capable of causing 50% hemolysis (IC_50_) in human erythrocytes. The experiment was performed in triplicate at two different time points.

#### 2.6.2. Mutagenicity Test

The mutagenic potential of AES was evaluated using the Ames test, following the protocol by Kado et al. [43]. The assays were conducted with and without the S9 microsomal fraction (Moltox®, Boone, NC, USA), utilizing the *Salmonella* Typhimurium strains TA98 and TA100 (Moltox®, Boone, NC, USA). In test tubes, 50 µL of 0.2 M phosphate buffer or S9 fraction, 5 µL of AES at concentrations of 50, 150, 500, 1500, and 5000 µg/plate, and 50 µL of bacterial suspension were added. The mixture was pre-incubated at 37 °C for 90 min, followed by the addition of 2 mL of top agar, which was poured onto minimal agar plates. The plates were incubated at 37 °C for 48–66 h, and subsequently, the revertant colonies were counted.

Positive controls without metabolic activation included 4-nitro-o-phenylenediamine (Merck KGaA, Darmstadt, Germany) (TA98) and sodium azide (Merck KGaA, Darmstadt, Germany) (TA100). For metabolic activation, 2-aminoanthracene (Merck KGaA, Darmstadt, Germany) was used for both strains. Distilled water was used as a negative control. To indicate mutagenic potential, three criteria were considered: mutagenic index (MI) ≥ 2, presence of a dose–response relationship, and statistical significance. Samples with MI ≤ 0.7 were considered potentially toxic, according to Kummarow et al. [44]. The experiment was performed in triplicate at two different time points.

### 2.7. Selectivity Index (SI)

The SI values were determined by the ratio of the IC_50_ from the hemolytic activity assay to the MIC (IC_50_/MIC) of AES against the reference strain *C. albicans* ATCC 90028 and clinical isolates of *C. albicans*, indicating the specificity of AES for microorganisms versus human cells. An SI > 1 indicates selectivity of the extract, while values < 1 indicate non-selectivity [45].

### 2.8. Statistical Analysis

The results were statistically analyzed using ANOVA followed by Tukey’s test. Differences were considered statistically significant at *p* < 0.05. Statistical analyses, graphs, and determination of IC_50_ were performed using the software GraphPad Prism^®^ 9.0 (GraphPadSoftware, San Diego, CA, USA).

## 3. Results

### 3.1. Chemical Characterization

**Figure A1:**
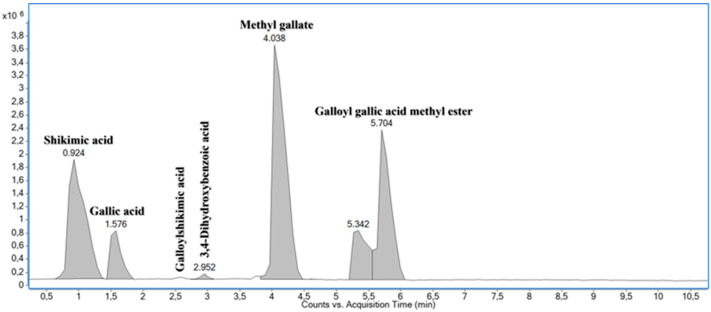
Full scan base peak chromatogram (BPC) of the AES in the negative ESI mode.

**Figure A2:**
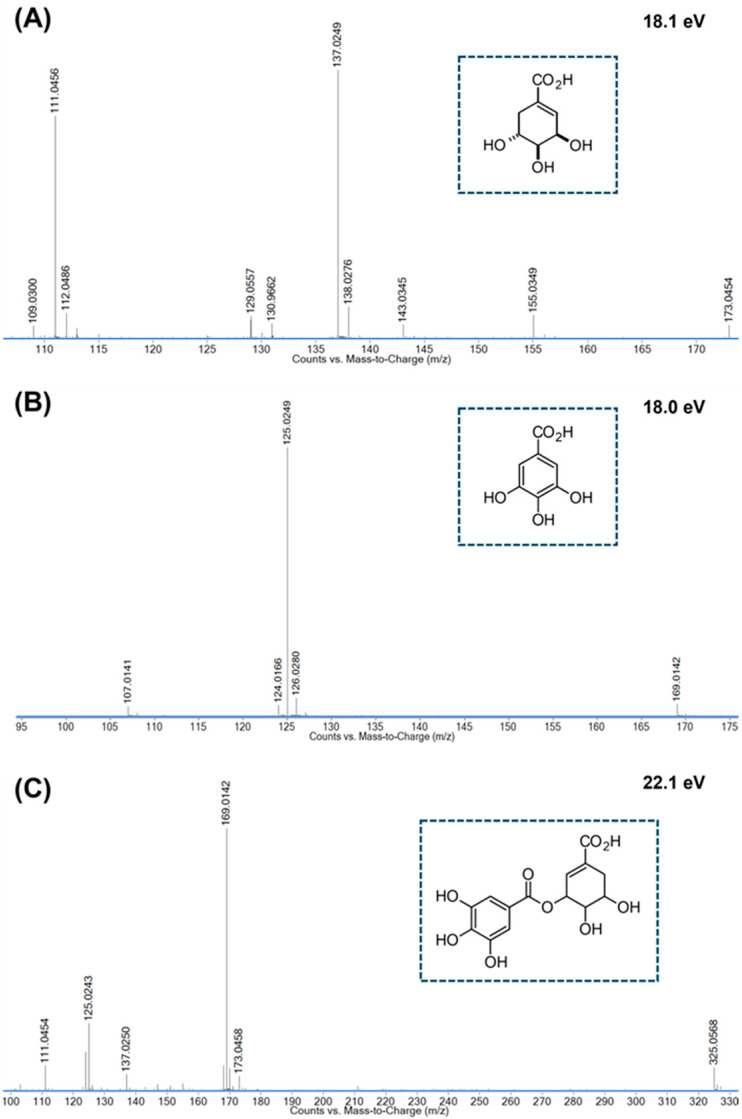
ESI(-)-MS/MS (precursor ion: [M-H]^−^) spectra obtained for shikimic acid (**A**), gallic acid (**B**), and galloylshikimic acid* (**C**) identified in AES. * Connectivity depicted is illustrative only.

**Figure A3:**
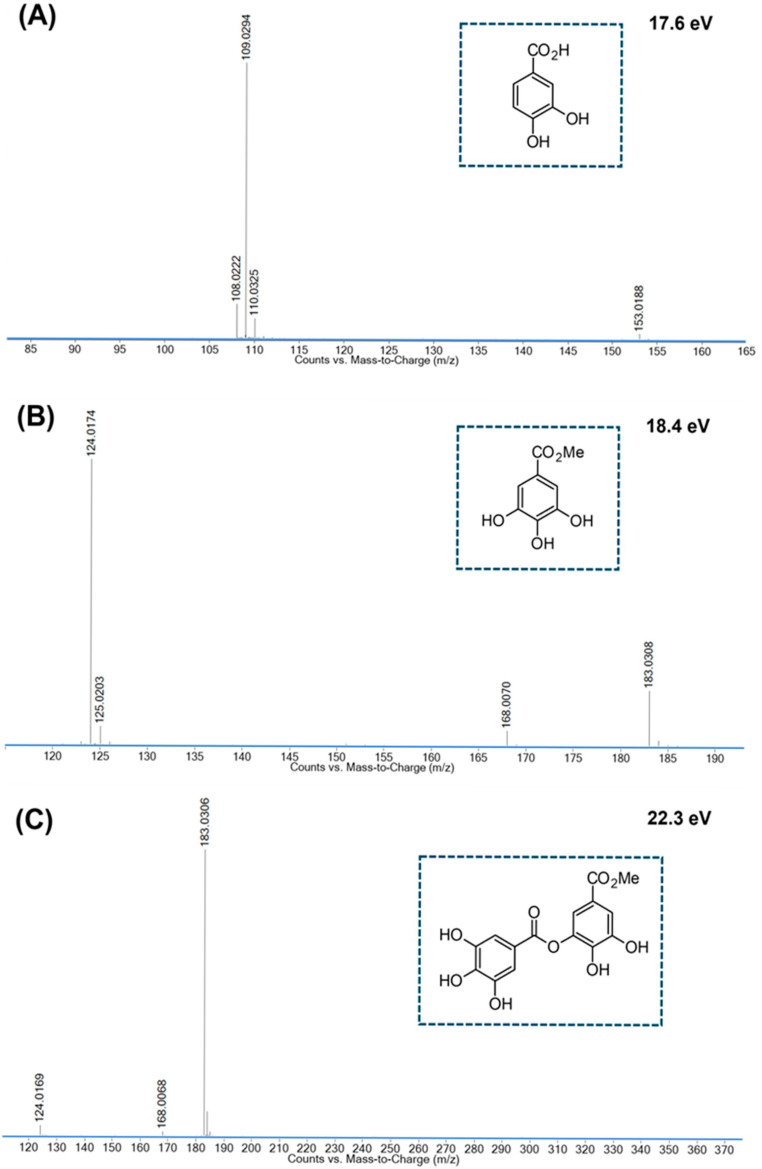
ESI (-)-MS/MS (precursor ion: [M-H]^−^) spectra obtained for 3,4-dihydroxybenzoic acid (**A**), methyl gallate (**B**), and galloyl gallic acid methyl ester* (**C**) identified in AES. * Connectivity depicted is illustrative only.

The aqueous extract of leaves of *Schinus weinmanniifolia* Mart. ex Engl. (AES) showed a yield of 16% in its aqueous extraction. AES was found to predominantly contain compounds that are derived from the shikimate pathway (Table 1; Figure A1, Figure A2 and Figure A3 in Appendix A). A total of six compounds, including a gallic acid-shikimic acid hybrid (compound 3 in Table 1) and depside (Compound 6 in Table 1), were annotated for the AES. The putative chemical structures of these compounds were proposed based on accurate *m/z* values of cationized and/or anionized molecules, interpretation of MS/MS data, and comparisons of the MS and MS/MS data obtained with those available in the literature [33,46,47,48]. Fragmentation data supporting the annotations of the compounds as shikimic acid, gallic acid, galloylshikimic acid, 3,4-dihydroxybenzoic acid, methyl gallate, and galloyl gallic acid methyl ester, respectively, are provided in Table 1.

Compound 1 was detected as a deprotonated molecule with an *m/z* 173.0450, displaying a fragmentation pattern consistent with that reported for shikimic acid [47]. As expected for this polyhydroxylated carboxylic acid, with molecular formula C_7_H_10_O_5_, the ESI(-)-MS/MS spectrum of 1 showed peaks at *m/z* 155.03, 137.02, and 111.04. These diagnostic peaks for shikimic acid were attributed to anionic fragments formed by the sequential loss of two H_2_O molecules or the loss of a H_2_O molecule followed by CO_2_ elimination. The loss of H_2_CO was also observed, further supporting the presence of hydroxyl groups in 1. The annotation of 1 as shikimic acid was further supported by comparing its ESI(-)-MS/MS spectrum and retention time with those of an authentic standard analyzed on the same analytical platform.

The molecular formulas of compounds 2 and 4 were determined to be C_7_H_6_O_5_ and C_7_H_6_O_4_, respectively, by high-resolution mass spectrometry (HRMS). Regardless of the ionization mode (positive or negative), both compounds fragmented by eliminating essentially the same neutral fragments. The acidic natures of 2 and 4 were proposed based on the elimination of a CO_2_ molecule from the respective [M-H]^−^ ions, which was found to be the main fragmentation pathway in both cases. The presence of a carboxyl group in 2 and 4 was further supported by the peaks at *m/z* 153.02 and 137.02. These peaks were observed in the ESI(+)-MS/MS spectra of 2 and 4, respectively, and attributed to the acyllium cations [(3,4,5-trihydroxyphenyl)methylidyne] oxidanium and [(3,4-dihydroxyphenyl)methylidyne]oxidanium. Thus, compounds 2 and 4 were found to be the benzoic acid derivatives gallic and 3,4-dihydroxybenzoic acids, respectively.

The ESI(+)-MS/MS and ESI(-)-MS/MS spectra of compound 5 closely resembled those of compound 2. However, the deprotonated and protonated molecules generated from 5 were 14 u higher than those from 2. This difference in the *m/z* value indicated that compound 5 was a monomethylation product of compound 2, which was corroborated by the detection of a radical anion fragment ([M-CH3-H]^•−^) with *m/z* 168.01 in the negative ionization mode. The presence of a carbomethoxy group in 5, rather than an aromatic methoxy group, was suggested by the loss of a CH_3_OH molecule (−30 Da) from its conjugated acid. This type of α-cleavage is common in the fragmentation of methyl esters and is well-documented in the literature [49]. Consequently, compound 5 was proposed to be the methyl ester of 2 and annotated as methyl gallate.

Compound 3 was detected as [M+Na]^+^, [M+H]^+^, and [M-H]^−^ ions of *m/z* 349.0539, 327.0731, and 325.0567, respectively. Hence, its molecular formula was established as C_14_H_14_O_9_, indicating eight degrees of unsaturation. In the negative ionization mode, compound 3 fragmented to give the conjugated bases of 1 and 2 (fragment ions with *m/z* 173.04 and 169.01, respectively), along with the main diagnostic fragment ions observed for 1 (*m/z* values: 137.02 and 111.04) and 2 (*m/z* value: 125.02). Based on this information, compound 3 was proposed to be a gallic acid–shikimic acid hybrid, with the connectivity between the galloyl and shikimate moieties yet to be determined. Consequently, the connectivity shown in Figure A3 is provisional and illustrative only.

Compound 6 was determined to have the molecular formula C_15_H_12_O_9_, consistent with the *m/z* values found for the respective [M+H]^+^ and [M-H]^−^ ions (Table 1). In the ESI(-)-MS/MS spectrum of 6, only peaks at *m/z* 183.03, 168.01, and 124.02 were observed. These peaks matched those verified in the ESI(-)-MS/MS spectrum of 5, providing strong evidence in favor of a methyl galloyl moiety in 6. The presence, in turn, of a galloyl moiety in the chemical structure of 6 was proposed based on the characteristic loss of a neutral fragment of 152 Da, which was verified in the negative ionization mode and is consistent with previous reports on the fragmentation of hydrolyzable tannins and depsides [48].

### 3.2. Antioxidant Activity

The IC_50_ values for AES and the positive controls are shown in Table 2. In the DPPH free radical scavenging assay, AES exhibited an IC_50_ of 5.51 µg/mL, with no statistically significant difference compared to BHT (*p* > 0.05). In the ABTS free radical scavenging assay, AES achieved an IC_50_ of 1.52 µg/mL, which is 21% lower than that of AA (IC_50_ = 1.92 µg/mL) and 77% lower than that of BHT (IC_50_ = 6.62 µg/mL).

### 3.3. Antifungal Activity

#### 3.3.1. Minimum Inhibitory Concentration (MIC)

For *Candida* species (ATCC), *N. glabrata* and *P. kudriavzevii*, the MIC value of AES ranged from 0.48 to 1.95 µg/mL. For *Cryptococcus* species the values ranged from 0.97 to 62.5 µg/mL (Table 3). In evaluating AES for the control of clinical isolates of vulvovaginal candidiasis (VVC), the MIC value for all clinical isolates was 1.95 µg/mL, consistent with the reference strain *C. albicans* ATCC 90028 (Table 3).

#### 3.3.2. Growth Kinetics of *C. albicans*

**Figure 1:**
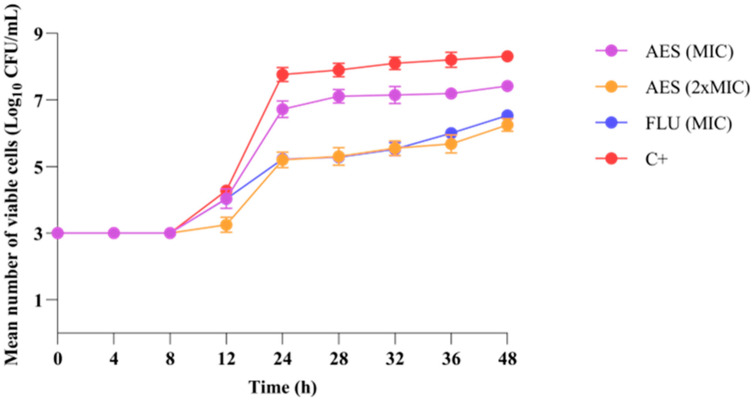
Activity of aqueous extract of leaves of *S. weinmanniifolia* (AES) on the growth kinetics of *C. albicans* ATCC 90028. MIC: minimum inhibitory concentration. 2 × MIC: two-fold the minimum inhibitory concentration. FLU: fluconazole. C+: positive control (cells in culture medium). CFU: colony forming unit. Statistically significant difference (*p* < 0.05) two-way ANOVA was followed by Tukey’s post hoc test.

The effect of AES on the growth kinetics of *C. albicans* ATCC 90028 (Figure 1) was evaluated in a time curve (0–48 h), which indicated that, up to 8 h, all tested samples showed no statistically significant difference (*p* > 0.05). At the 12 h incubation time, 2 × MIC of AES showed a significant reduction in fungal growth compared to the positive control (*p* < 0.05). From 24 to 48 h, AES at 2 × MIC resulted in a 2 log_10_ in viable cell count relative to the positive control. During the same period, 2 × MIC of AES did not show a statistically significant difference in relation to the MIC of FLU (*p* > 0.05).

### 3.4. Mechanisms of Action in Planktonic Cells of C. albicans

#### 3.4.1. Scanning Electron Microscopy (SEM)

**Figure 2:**
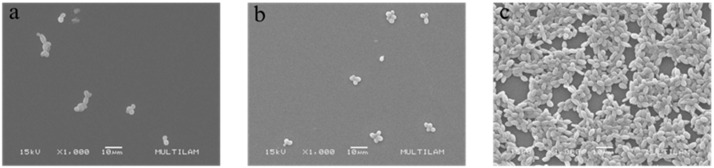
Scanning electron microscopy (1000× magnification) images showing the morphology and cellular organization of *C. albicans* ATCC 90028 treated and untreated with the aqueous extract of leaves of *S. weinmanniifolia* (AES). (**a**) Minimum inhibitory concentration of AES. (**b**) Two-fold the minimum inhibitory concentration of AES. (**c**) Positive control (*C. albicans* cells in culture medium without AES treatment).

SEM analysis revealed that both concentrations of AES (MIC and 2 × MIC) evaluated substantially reduce the number of *C. albicans* cells. No morphological alterations suggestive of structural damage were observed in the treated cells (Figure 2).

#### 3.4.2. Germ Tube Formation

**Figure 3:**
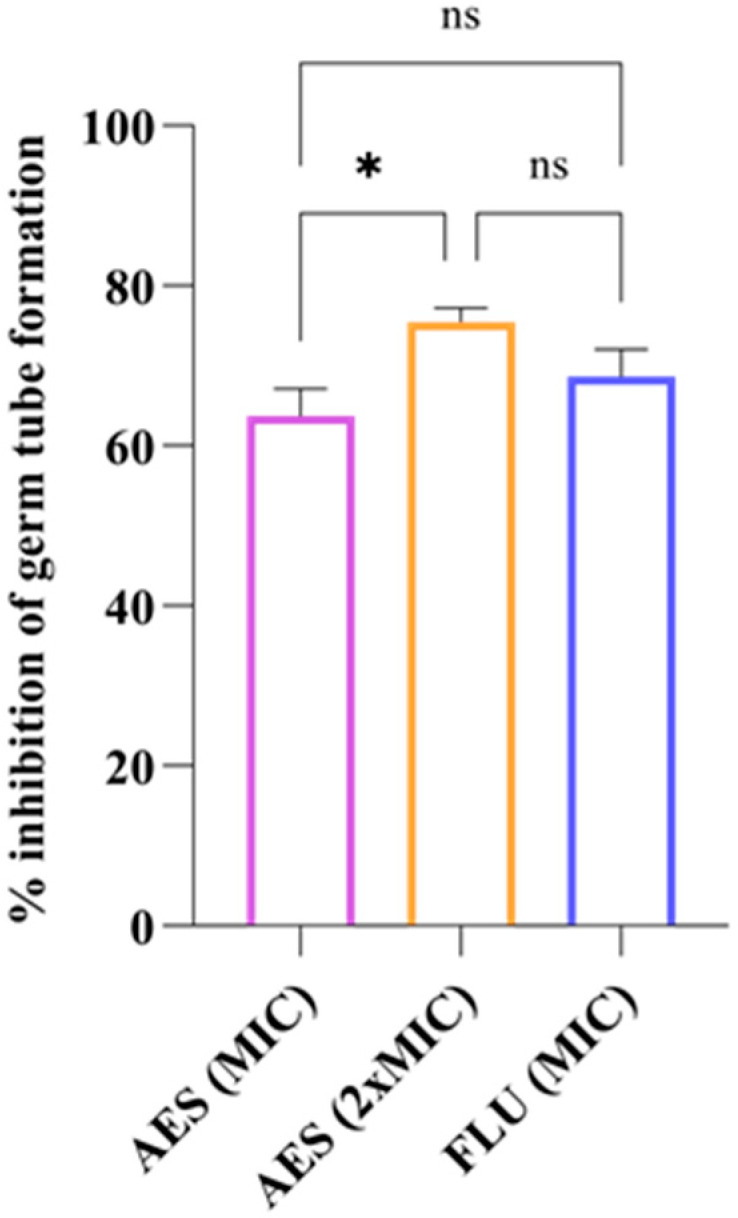
Effect of aqueous extract of leaves of *S. weinmanniifolia* (AES) on germ tube formation in *C. albicans* ATCC 90028. MIC: minimum inhibitory concentration. 2 × MIC: two-fold the minimum inhibitory concentration. FLU: fluconazole. Data are presented as means, with error bars indicating standard deviation. One-way ANOVA was performed followed by Tukey’s post hoc test, with *: *p* = 0.0367 (statistically significant difference) and ns: *p* > 0.05 (not significant).

AES inhibited germ tube formation in *C. albicans* ATCC 90028 by 63.72% at the MIC and 75.49% at the 2 × MIC. The inhibition rate achieved by FLU was 68.63% (Figure 3). There was no statistically significant difference between the two concentrations of AES when compared to FLU (*p* > 0.05).

#### 3.4.3. Yeast-to-Hyphal Transition

**Figure 4:**
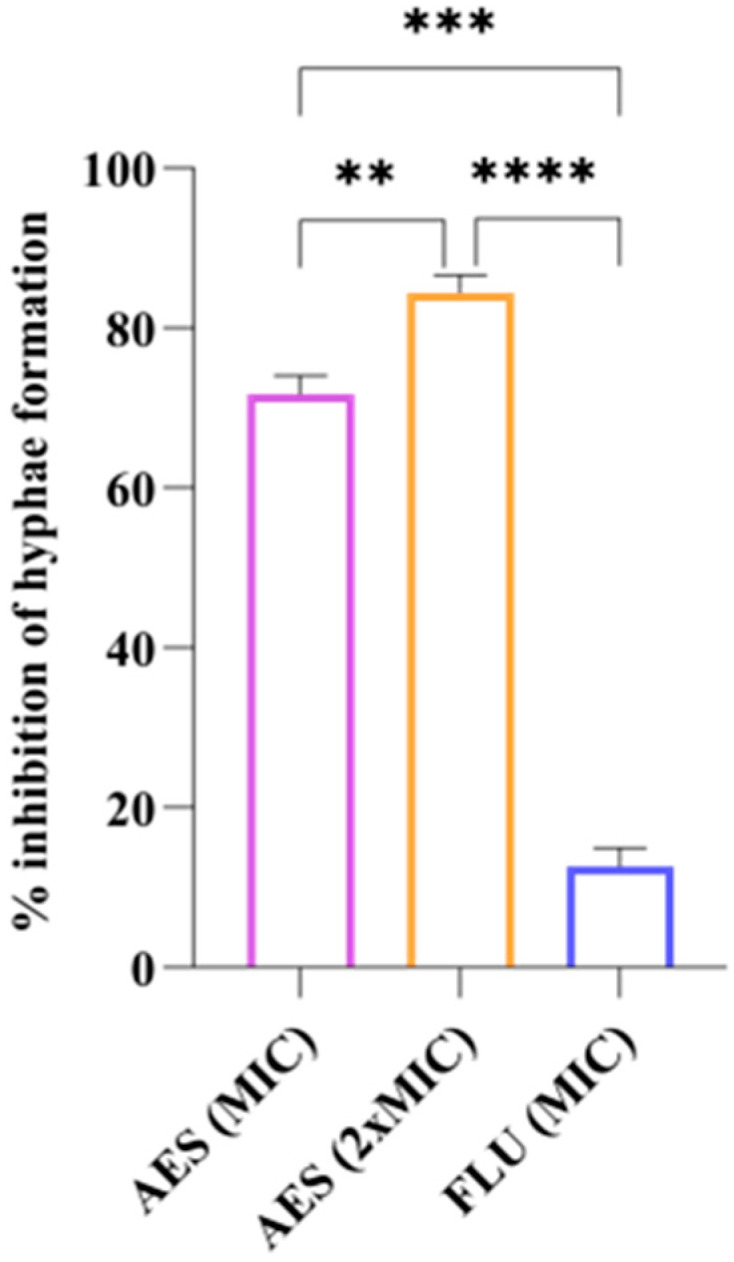
Effect of aqueous extract of leaves of *S. weinmanniifolia* (AES) on hyphae formation in *C. albicans* ATCC 90028. MIC: minimum inhibitory concentration. 2 × MIC: two-fold the minimum inhibitory concentration. FLU: fluconazole. Data are presented as means, with error bars indicating standard deviation. One-way ANOVA was performed followed by Tukey’s post hoc test, with statistically significant difference **: *p* = 0.0023; ***: *p* = 0.0005; ****: *p* = 0.0001.

Figure 4 shows the inhibitory effect of AES on the hyphal formation in *C. albicans* ATCC 90028. The 2 × MIC concentration of AES demonstrated the highest inhibition rate (84.34%), followed by MIC of AES (71.75%) and FLU (12.62%). All treatments exhibited statistically significant differences (*p* < 0.05).

### 3.5. Biocompatibility

#### 3.5.1. Hemolytic Activity

At all concentrations tested (0.24 to 1000 µg/mL), AES exhibited no hemolytic activity in human erythrocytes, with an IC_50_ > 1000 µg/mL. The highest concentration tested is 512.8-fold greater than the MIC of AES for *C. albicans* (ATCC 90028 and clinical isolates).

#### 3.5.2. Mutagenicity Test

Table 4 presents the results of the mutagenicity assessment of AES. At all concentrations tested (50 to 5000 μg/mL), no mutagenicity index was observed (MI > 2), and no cytotoxic profile was detected (MI < 0.7), both in the presence and absence of metabolic activation. The highest concentration tested is 2564 -fold greater than the MIC of AES for *C. albicans* (ATCC 90028 and clinical isolates).

### 3.6. Selectivity Index (SI)

The ratio between the IC_50_ for hemolytic activity in human erythrocytes and the MIC against *C. albicans* (ATCC 90028 and clinical isolates) for AES indicated a high selectivity index of 512.82. This result underscores the selective antifungal potential of AES, demonstrating significant activity against fungal cells while exhibiting low toxicity to human cells.

## 4. Discussion

RVVC constitutes a public health issue with global progression and presents high prevalence rates in Latin American countries such as Argentina, Brazil, Bolivia, Uruguay, and Venezuela [3]. In this context, reports from the Pan American Health Organization (PAHO) highlight the socioeconomic inequalities and disparities in access to healthcare faced by Latin American women [5,6]. Studies by Morales-Ramírez et al. [9] and Jansåker et al. [8] document situations of social vulnerability among patients with VVC in Latin America. Within this scenario, PAHO also emphasizes the limited access to healthcare centers for women in various Latin American countries and underscores the importance of knowledge and use of ancestral medicines [7].

The search for new complementary treatment options through natural resources represents a promising strategy to reduce recurrences, prevent severe cases [50], and, most importantly, support vulnerable populations facing limitations in accessing conventional therapies. The native occurrence of *S. weinmanniifolia* in developing countries, combined with the scarcity of studies on this species, reinforces the need to investigate its bioactive properties in order to guide future applications in traditional medicine.

In the past decade, the strengthening of green chemistry has gained increasing prominence, focusing on renewable techniques that cause less impact on humans and the environment, such as the use of water as a solvent, due to its greater safety and accessibility [51]. Aqueous extraction is the method that most closely aligns with the traditional context of popular use, as the preparation and consumption of plant infusions, whether orally or through topical application, are timeless practices. It was observed that, compared to the yield of the ethanolic extract from *S. weinmanniifolia* leaves (6.32%) described by Ferreira et al. [32], the aqueous extract presents a yield 2.5 times higher.

From the perspective of the biological activities of AES, its antioxidant potential was investigated due to the association between oxidative stress, antioxidant depletion, and VVC. In the study conducted by Heydarian Moghadam et al. [14], 95 women with VVC used a gel containing *Boswellia serrata* plant extract. Analyses of vaginal secretions demonstrated an increase in total antioxidant capacity (TAC), a reduction in the oxidative stress marker malondialdehyde (MDA), decreased expression of Bax and Casp3 (cell apoptosis markers), and increased expression of Bcl2 (a cellular protection marker), which resulted in the minimization of infection symptoms.

AES, when evaluated using two distinct free radical scavenging techniques, demonstrated antioxidant activity classified as very strong, according to the descriptions by Wahyuni et al., for extracts with IC_50_ < 50 μg/mL [52]. This potential may be associated with the presence of gallic acid, which contains a tri-hydroxylated phenolic structure at positions 3, 4, and 5 of the benzene ring, along with a carboxyl group. The antioxidant activity of this compound is related to hydrogen atom transfer (HAT) and single electron transfer (SET) mechanisms [53].

AES demonstrated promising antifungal potential in controlling the growth of clinically relevant yeasts. Despite being a crude extract without prior compound isolation, AES exhibited the best MIC compared to values reported in the literature for crude extracts, fractionated extracts, and isolated compounds [54], as well as drug-like molecules [55] that applied the interpretation of a 50% reduction in fungal growth in assays with *C. albicans*. In their research, Alves et al. [56] conducted a comprehensive survey considering the MIC of synthesized molecules, classifying samples with MIC < 3.515 μg/mL as having ”very strong bioactivity”. AES fits this classification based on the MIC values obtained against *C. albicans* (reference strain and clinical isolates), *C. tropicalis*, *C. parapsilosis*, *N. glabrata*, *P. kudriavzeveii*, and *C. neoformans*.

The time-growth binomial analysis showed that 2 × MIC of AES acted in controlling the development of *C. albicans* starting at 12 h of contact, whereas the MIC of FLU began its action at 24 h. Samples capable of acting on planktonic *C. albicans* cells during the early stages of morphological transition, that is, within the first 12 h [57], stand out for reducing the microbial population size, which may contribute to preventing biofilm formation and controlling virulence processes. Thus, 2 × MIC of AES proves to be promising from the first hours of contact with *C. albicans*, while maintaining logarithmic growth control that does not differ statistically from the MIC of FLU in the 24–48 h period. According to the classification proposed by Klepser et al. [38], AES is classified as fungistatic, as is FLU, since both present growth greater than 3 log_10_ at the end of the analysis.

When exposed to favorable conditions, such as increased temperature and humidity, the presence of nutrients, and neutral or alkaline pH, *C. albicans* transitions from its yeast form (blastoconidia) and begins forming germ tubes, which are elongated, non-septate structures. This initial stage is a virulence marker and leads to the development of true hyphae, promoting tissue penetration, resistance to phagocytosis, biofilm formation, and enhanced virulence, particularly in relation to vaginal inflammation [58,59,60].

SEM analysis revealed that AES is capable of reducing the number of *C. albicans* cells without causing damage to the cell wall. Additionally, AES significantly reduced the formation of germ tubes and hyphae in *C. albicans*. Substances that act on the morphogenesis of *C. albicans* have been considered promising therapeutic options, as interfering with specific virulence mechanisms is advantageous for preventing progression to more severe stages of recurrent infection, such as biofilm formation, and for avoiding the selective pressure that leads to fungal resistance [61]. The action of AES on planktonic cells and virulence factors of *C. albicans* may be associated with the presence of gallic acid in its composition, since this compound has been shown to reduce the formation of germ tubes [62] and hyphae in *C. albicans* [63].

In addition to its promising antifungal activity, AES stands out for its biocompatibility. The cytotoxicity assessment in human erythrocytes is a sensitive technique that indicates the sample’s potential to cause cell membrane rupture, resulting in the release of hemoglobin into the extracellular medium. According to the classification by Costa-Lotufo et al. [64] for hemolytic activity assays, plant extracts with IC_50_ > 200 μg/mL are considered non-hemolytic, as demonstrated by the AES results, even when tested at high concentrations.

In addition to cytotoxicity screening through the hemolysis assay, the Ames test contributes to the assessment of parameters associated with mutagenicity, enabling the detection of the potential to induce gene mutations in new drug candidates, environmental samples, and plant extracts [65]. This test stands out as one of the key steps in preclinical assays to ensure the safety of therapeutic products. Therefore, regulatory agencies such as the United States Food and Drug Administration (FDA), the European Medicines Agency (EMA), and the Agência Nacional de Vigilância Sanitária (ANVISA) recommend the Ames test as a mandatory step in the battery of assays to prevent the release of substances with mutagenic potential [65]. The absence of mutagenicity observed for AES, both in the presence and absence of exogenous metabolic activation, reinforces the potential of this extract as a therapeutic input and encourages future studies focused on evaluating its pharmacological safety. This interest is further justified by the high selectivity index demonstrated, which may contribute to safer and more targeted popular use.

Thus, from a traditional popular use perspective, AES is characterized as a promising candidate for future studies evaluating its application in sitz baths (hydrotherapy), a technique that involves immersing the pelvic region in water which may contain medicinal herbs, essential oils, or drugs, aiming to alleviate uterine cramps, hemorrhoids, vaginal infections, swelling, and itching in the genital area [66]. This is a common and accessible practice among women in vulnerable situations who do not have access to conventional drug therapies. In this context, *S. terebinthifolius* has traditionally been recommended for such use. The use of AES in the early stages of RVVC could contribute to controlling *C. albicans* in its planktonic phase, resulting in reduced fungal burden and inhibition of hyphae formation, thereby supporting the host’s defense mechanisms [59].

## 5. Conclusions

This study presents the first report on the chemical composition and antifungal properties of the aqueous extract of *S. weinmanniifolia* leaves, with a focus on its activity against *C. albicans* isolated from VVC. Thus, it is important to highlight that: (i) aqueous extraction is easily reproducible, low-cost, and similar to traditional practices; (ii) AES demonstrated promising activity against *C. albicans *(*including clinical isolates*); (iii) the action of AES may be associated with the inhibition of *C. albicans* virulence mechanisms, with potential for therapies targeting morphogenesis interference; (iv) even at high concentrations, AES did not exhibit cytotoxicity or mutagenicity profiles; (v) *Schinus weinmanniifolia*, a species native to Latin American countries, shows potential for popular use, especially among women in socially vulnerable groups and with limited access to healthcare services and conventional medications. Further studies investigating pharmacological safety, in vivo efficacy, topical bioavailability, and stability in aqueous formulations are warranted to support future therapeutic development.

## Figures and Tables

**Table 1:** Compounds annotated for the AES by means of UHPLC-ESI-MS/MS analyses (mass accuracy tolerance ±5 ppm).

No.	*t_R_* (min)	FM	Ionized Molecules and Relevant Fragment Ions (m/z)					Annotation
			MS	Error	MS	Error	MS/MS [+ (#) and − (*) modes]	[Reference(s)]
**1**	0.924	C_7_H_10_O_5_			347.0965 [2M-H]^−^ 173.0450 [M-H]^−^	−3.8 0.0 ***	*173.04 → 155.04; 143.03; 137.02; 111.05	Shikimic acid [47]
**2**	1.576	C_7_H_6_O_5_	171.0293 [M+H]^+^	0.0 **	169.0134 [M-H]^−^	−1.8	#171.03 → 153.02; 135.01; 125.02; 109.03; 107.01; * 169.01 → 125.02; 124.02; 107.01	Gallic acid [48]
**3**	2.590	C_14_H_14_O_9_	349.0539 [M+Na]^+^ 327.0731 [M+H]^+^	+1.0 +4.6	325.0567 [M-H]^−^	+2.3	#325.06 → 153.02; * 325.06 → 173.04; 170.02; 169.01; 168.01; 137.02; 125.04; 124.02; 111.04	Galloylshikimic acid [46]
**4**	2.952	C_7_H_6_O_4_	155.0347 [M+H]^+^	+1.7	153.0182 [M-H]^−^	−3.8	#155.03 → 137.02; 109.03; * 153.02 → 110.03; 109.03; 108.02	3,4-Dihydroxybenzoic acid [33]
**5**	4.038	C_8_H_8_O_5_	185.0455 [M+H]^+^	+2.7	367.0656 [2M-H]^−^ 183.0300 [M-H]^−^	−2.5 +3.6	#185.04 → 154.02; 153.02; 126.03; 125.02; 123.04; 107.01; * 183.03 → 168.01; 125.02; 124.02	Methyl gallate [46,48]
**6**	5.196–5.993	C_15_H_12_O_9_	337.0570 [M+H]^+^	+3.1	671.0865 [2M-H]^−^ 335.0402 [M-H]^−^	−2.9 −0.3	#337.06 → 153.02; 125.02; * 335.04 → 183.03; 168.01; 124.02	Galloyl gallic acid methyl ester [48]

Metabolite identification level: 2 (putative identification). # Fragment ions generated in the positive ionization mode. * Fragment ions generated in the negative ionization mode. ** The positive ionization mode was internally recalibrated using the measured and known *m/z* values of the [M+H]^+^ ion generated from compound **2**. *** The negative ionization mode was internally recalibrated using the measured and known *m*/*z* values of the [M-H]^−^ ion generated from compound **1**.

**Table 2:** Antioxidant activity (IC_50_) of aqueous extract of leaves of *S. weinmanniifolia* (AES) and standard antioxidants Ascorbic Acid (AA) and Butylated Hydroxytoluene (BHT).

	IC_50_ (µg/mL)	
Sample	DPPH	ABTS
AES	5.51 ± 0.25 ^b^	1.52 ± 0.06 ^c^
AA	3.91 ± 0.28 ^a^	1.92 ± 0.08 ^a^
BHT	5.53 ± 0.14 ^b^	6.62 ± 0.15 ^b^

IC_50_: Mean concentration in µg/mL capable of inhibiting 50% of free radicals in the reaction ± standard deviation. DPPH: radical scavenging 2,2-diphenyl-1-picrylhydrazyl. ABTS: radical scavenging 2,2-azino-bis (3-ethylbenzothiazoline-6-sulfonic acid). AA: ascorbic acid. BHT: butylated hydroxytoluene. AES: aqueous extract of leaves of *S. weinmanniifolia*. Different letters vertically represent Statistically significant difference (*p* < 0.05) one-way ANOVA, was followed by Tukey’s post hoc test.

**Table 3:** Minimum inhibitory concentration (µg/mL) of aqueous extract of leaves of *S. weinmanniifolia* (AES) against the yeasts (ATCC) and vulvovaginal candidiasis isolates.

Microorganism	AES	FLU
*Candida albicans*	1.95	0.125
*Candida tropicalis*	0.97	0.125
*Candida parapsilosis*	0.97	1
*Nakaseomyces glabrata*	0.48	8
*Pichia kudriavzeveii*	0.48	-
*Cryptococcus gattii*	62.5	8
*Cryptococcus neoformans*	0.97	8
*C. albicans* CMRP3475	1.95	0.125
*C. albicans* CMRP3476	1.95	0.125
*C. albicans* CMRP3477	1.95	0.125
*C. albicans* CMRP3478	1.95	0.125
*C. albicans* CMRP3479	1.95	0.125

FLU: Fluconazole. AES: aqueous extract of leaves of *S. weinmanniifolia. Pichia kudriavzeveii* (*C. krusei*) is intrinsically fluconazole resistant (-).

**Table 4:** Mutagenic activity of aqueous extract of leaves of *S. weinmanniifolia* (AES) expressed by the mean number of revertant colonies/plate ± standard deviation and mutagenicity index against *S.* Typhimurium strains TA98 and TA100 in the absence (S9) and presence (S9+) of metabolic activation.

AES				
[µg/plate]	TA98		TA100	
	S9(−)	S9(+)	S9(−)	S9(+)
**0^a^**	52 ± 6	54 ± 8	88 ± 5	87 ± 5
**50**	48 ± 2 (0.9)	59 ± 1 (1)	123 ± 11 (1) *	119 ± 3 (1) **
**150**	44 ± 1 (0.8)	60 ± 3 (1)	145 ± 4 (1) **	115 ± 4 (1) **
**500**	43 ± 1 (0.8)	47 ± 6 (0.8)	157 ± 8 (1) **	108 ± 4 (1) *
**1500**	47 ± 3 (0.9)	45 ± 4 (0.8)	126 ± 3 (1) **	150 ± 5 (1) **
**5000**	47 ± 3 (0.9)	51 ± 4 (0.9)	117 ± 5 (1) **	138 ± 3 (1) **
**C+**	260 ± 9 ^b^	293 ± 7 ^c^	677 ± 9 ^b^	708 ± 7 ^d^

S9(−): absence of metabolic activation. S9(+): presence of metabolic activation. Negative Control (0^a^): DMSO. Positive Control (C+): ^b^ 4-nitro-o-phenylenediamine (10 μg/plate). ^c^ 2AA-aminoanthracene (2.5 μg/plate). ^d^ Sodium Azide (2.5 μg/plate). Significant difference (ANOVA): * *p* < 0.05; ** *p* < 0.01.

## Data Availability

The original contributions presented in this study are included in the article/Appendix A. Further inquiries can be directed to the corresponding author(s).

## Abbreviations

The following abbreviations are used in this manuscript:

AA	Ascorbic acid
ABTS	2,2-azino-bis (3-ethylbenzothiazoline-6-sulfonic acid)
AES	*Schinus weinmanniifolia* Mart. ex Engl.
ANVISA	Agência Nacional de Vigilância Sanitária
ATCC	American Type Culture Collection
BHT	Butylated hydroxytoluene
BPC	Base peak chromatogram
CFU	Colony Forming Unit
DPPH	2,2-diphenyl-1-picrylhydrazyl
EMA	European Medicines Agency
FDA	Food and Drug Administration
FLU	Fluconazole
GRP	Germination reduction percentage
GTF	Germ tube formation
HAT	Hydrogen atom transfer
HRMS	High-resolution mass spectrometry
IC50	Half-maximal inhibitory concentration
MDA	Marker malondialdehyde
MI	Mutagenic Index
MIC	Minimum Inhibitory Concentration
PBS	Phosphate-buffered saline
RVVC	Recurrent Vulvovaginal Candidiasis
SEM	Scanning electron microscopy
SET	Single electron transfer
SI	Selectivity index
TAC	Total Antioxidant Capacity
VVC	Vulvovaginal Candidiasis
YNB	Yeast Nitrogen Base
